# A Multi-Technology Communication Platform for Urban Mobile Sensing

**DOI:** 10.3390/s18041184

**Published:** 2018-04-12

**Authors:** Rodrigo Almeida, Rui Oliveira, Miguel Luís, Carlos Senna, Susana Sargento

**Affiliations:** 1 Instituto de Telecomunicações, 3810-193 Aveiro, Portugal; rui.filipe.oliveira@ua.pt (R.O.); nmal@av.it.pt (M.L.); cr.senna@av.it.pt (C.S.); susana@ua.pt (S.S.); 2 Departamento de Eletrónica, Telecomunicações e Informática, University of Aveiro, 3810-193 Aveiro, Portugal

**Keywords:** smart cities, Internet of Things, multi-technology communication, low-power wide-area networks, delay-tolerant network, urban sensor network

## Abstract

A common concern in smart cities is the focus on sensing procedures to provide city-wide information to city managers and citizens. To meet the growing demands of smart cities, the network must provide the ability to handle a large number of mobile sensors/devices, with high heterogeneity and unpredictable mobility, by collecting and delivering the sensed information for future treatment. This work proposes a multi-wireless technology communication platform for opportunistic data gathering and data exchange with respect to smart cities. Through the implementation of a proprietary long-range (LoRa) network and an urban sensor network, our platform addresses the heterogeneity of Internet of Things (IoT) devices while conferring communications in an opportunistic manner, increasing the interoperability of our platform. It implements and evaluates a medium access communication (MAC) protocol for LoRa networks with multiple gateways. It also implements mobile Opportunistic VEhicular (mOVE), a delay-tolerant network (DTN)-based architecture to address the mobility dimension. The platform provides vehicle-to-everything (V2X) communication with support for highly reliable and actionable information flows. Moreover, taking into account the high mobility pattern that a smart city scenario presents, we propose and evaluate two forwarding strategies for the opportunistic sensor network.

## 1. Introduction

Technological progress over the recent decades, favored by developments in information and communication technology (ICT), has revolutionized the way people live their everyday lives. As a result, paradigms such as Internet of Things (IoT) have emerged. IoT is a paradigm where any everyday object can be equipped with both processing and communication capabilities in order to collect and exchange information between *things*, or the Internet [1]. By enabling interaction with such wide and diverse devices, this paradigm finds applications in many different domains. One domain of particular interest is the urban context, since the paradigm tries to address the problems caused by the rapid increase in population density. To confront this adversity, the availability of public resources as well as the quality of services offered to the citizens should increase, while the operational costs of public administration should tend to decrease, encouraging the fast deployment of the smart city concept [2,3]. The smart city concept is the combination of the IoT paradigm in an urban context with the exploitation of ICT solutions [2]. Several core areas are covered by this paradigm. The smart environment [4,5] is one of them: it tries to address concerns regarding environmental protection, lack of energy efficiency, poor usage of the natural resources, environmental pollution, and sustainable resource management, among others.

A typical smart city scenario has to deal with an extensive number of sensors and data generators (some of which are placed in high mobile devices), deployed to collect and generate all types of information, which will increase the numbers of communicating machines, modifying the current scenario of human-centric communications. The consequence is an avalanche of mobile and wireless traffic information. The high heterogeneity and volatility of the network carries connectivity issues, such as long and variable delays, sparse and intermittent connectivity, high error rate, high latency, highly asymmetric data rate, or even a non-existent end-to-end connectivity [6]. Along with the necessity to have a low-cost infrastructure, and to overcome the issues associated with the network disruption, the concept of the delay-tolerant network [7] is usually adopted in these scenarios.

Moreover, in a smart city environment, a large number of devices are battery-powered and located in remote areas where wired connectivity is hard to guarantee. However, these devices need to be connected to cloud applications that offer a broad vision of city management. Low-Power Wide-Area Network (LPWAN) technology can be a good option to meet these requirements [8]. The long-range (LoRa) network is also a relevant form of LPWAN technology due to its unique modulation [9], which makes it a very versatile form of technology that can be easily adapted to different types of environments and applications [10]. Besides, it is seen as an attractive solution for IoT and machine-to-machine (M2M) platforms since it operates in unlicensed bands.

In this context, this work implemented and evaluated, through a real environment, a multi-technology opportunistic platform for environmental data gathering with respect to smart cities, considering both static and moving sensing elements, and both long- and short-range communication. The main contributions of this work can be summarized as follows:

- The evaluation of a novel medium access control (MAC) protocol for LoRa networks with multiple gateways;
- The evaluation of two urban-centric forwarding decision strategies for an opportunistic delay-tolerant network supporting a mobile sensing network;
- The joint evaluation of long- and short-range communications granting the possibility of collecting heterogeneous information in a smart city;
- Conclusions about the feasibility of each platform’s component, along with the overall platform performance.

The remainder of this paper is organized as follows. Section 2 discusses the relevant related work. Section 3 overviews the platform architecture, describing the network elements and functionalities. Section 4 presents the mechanisms supporting the multi-technology communication. Finally, Section 5 discusses the performance results and Section 6 enumerates the conclusions and future work.

## 2. Related Works

### 2.1. IoT Platforms for Smart Cities

Various experimental platforms have been implemented and assessed in order to evaluate their behavior and sustainability for several IoT applications through the employment of different communication technologies. This subsection aims to provide general information about some of the currently available testbeds for Internet of Things (IoT) experimentation and development.

Sanchez et al. [11] presented a large deployed smart city real implementation in the city of Santander, Spain, known as the SmartSantander project (http://www.smartsantander.eu/). SmartSantander proposes a city-scale experimental European research facility for the experimentation of architectures, key enabling technologies, services, and applications for the IoT context of a smart city by recurring to communication technologies such as Institute of Electrical and Electronics Engineers (IEEE) 802.15.4 as well as WiFi, 3G, Bluetooth, and Ethernet. Currently, it encompasses more than 10,000 diverse IoT devices (fixed and mobile sensor nodes, near-field communication (NFC) tags, gateway devices, citizens’ smartphones, etc.) [12].

Lanza [13] present a large-scale deployment of IoT devices on vehicles that are continuously driving around the city. This was carried out under the SmartSantander testbed. Also, the authors introduce three mobile sensing network strategies used for distributing the data gathered, namely periodic reporting through mobile broadband network, opportunistic vehicle-to-infrastructure (V2I) networking on top of IEEE 802.15.4 links, and the delay-tolerant network (DTN) approach using IEEE 802.11.

Latre et al. [14] presented the City of Things testbed. It is a multi-technology testbed which allows for the testing of novel smart city experiments (e.g. evaluation of network protocols, data gathering mechanisms) with a real large-scale deployment. The testbed is built within the city of Antwerp in Belgium. A major distinguishing factor of this testbed is the fact that it allows a wide range of wireless technologies, including WiFi, DASH7 (a form of LPWAN technology), Bluetooth Low-Energy, IEEE 802.15.4, and LoRa, among others. It allows connections with high and low bit-rate sensors at close and long range, respectively. Furthermore, the authors employed a use case regarding air quality measurements in the city of Antwerp [15]. They developed a real time demo on the City of Things architecture, consisting of a set of air quality sensors mounted on the roofs of cars with wireless capabilities such as LoRa, Sigfox, and DASH7. These radios allow for real-time streaming of data on three separate communication channels. Moreover, WiFi is used to download all data gathered during the day in bulk.

Luis et al. [16] presented the UrbanSense platform deployed in the city of Porto in Portugal. Aiming to collect relevant sensory data for a smart city environmental monitoring, this platform has a data-collecting unit (DCU) as its fundamental element. Data gathered by DCUs can be carried to the UrbanSense server through different methods: metropolitan fiber ring backhaul, cellular network backhaul, or through a deployed vehicular delay-tolerant network provided by buses and municipality vehicles equipped with WiFi hotspots. The different forwarding data mechanisms provide a wide range of applications since both real-time and delay-tolerant communications are considered.

Petrić et al. [17] described the LoRa Fabian as a network protocol stack and experimental network setup, which was deployed over the city of Rennes in France. Such experimental setup is able to generate traffic similar to a real IoT application such as sensor monitoring. This not only gives the possibility of extracting basic performance metrics like the packet error rate, but also metrics related to the LoRa physical layer. Thus, this experimental setup provides insights about the performance and evaluation methods of LoRa networks.

With the monitoring and managing of urban air pollution in mind, Li et al. [18] deployed a network of air quality sensors through static (fixed locations) and mobile installations (on top of trams) within the city of Zurich in Switzerland. The data collection is performed using the Global System for Mobile Communication (GSM).

With the aim of handling the solid waste management in a city, Bharadwaj et al. [19] proposed a complete IoT-based system to process the tracking, collecting, and monitoring of the solid waste in an automated and efficient manner. This system implies that each garbage bin is equipped with two infrared (IR) sensors at the middle and top of the bins to detect the level of garbage collected, a weight sensor at the bottom of the bins, a gas sensor to detect harmful gases, and a microcontroller equipped with LoRa communication capabilities in order to collect sensory data and forward it to a LoRa gateway. The system also considers the possibility of introducing further environmental sensors.

Similar to the previous IoT system, Saravanan et al. [20] presented a similar approach for water grid management. This system was implemented in Mori village situated in the south-eastern delta of Andhra Pradesh, India. Recurring to water quality sensors and LoRa capable nodes, this system employs an alert triggering mechanism in which various alerts can be triggered to alarm the authorities in case of any changes in water quality or flow.

Table 1 summarizes the core characteristics of the aforementioned IoT platforms and correlates them with our proposed platform.

Therefore, this work proposes a multi-technology platform (with short- and long-range communications) for data gathering over smart cities, where the implementation of a proprietary multi-gateway MAC protocol for LoRa, along with different forwarding mechanisms over an opportunistic and delay-tolerant network, are evaluated.

### 2.2. MAC in LoRa

Within smart city scenarios and IoT applications the network nodes operate under shared-medium conditions, i.e., the nodes will have to compete over a shared common communication channel. Thus, an efficient MAC scheme is an essential requirement to grant a successful operation of the network under such conditions. Wireless sensor network (WSN) and IoT applications require the fulfillment of some specifications in terms of the MAC protocol, such as multi-hop communication, resilience, and sometimes low-latency, among others [21]. Moreover, it is essential to provide fair access to the communication channel and avoid possible packet collisions. For Low-Power Wide Area Network (LPWAN) technologies, besides the underlying radio frequency characteristics, much of the technology value consists in satisfying the end user requirements such as the ability to create a network, control it, and offer bi-directional data flow.

Long-range (LoRa) technology can be supported by the LoRaWAN (LoRa Wide-Area Network), a MAC protocol based on the Aloha method. This MAC layer is very lightweight and essentially implements pure-Aloha with Listen-Before-Talk (LBT), resulting in low channel utility under high traffic load due to packet collisions [22]. Promoted by the LoRa Alliance, LoRaWAN [23] defines the communication protocol and system architecture for the LoRa physical (PHY) layer. This protocol and network architecture has the most influence in determining the battery lifetime of a node, the network capacity, the quality of service, the security, and the variety of applications served by the network. The LoRaWAN architecture is typically laid out in a star-of-stars topology in which gateways act as connection bridges between end-devices and a central network server (*NetServer*) in the backend. Gateways are connected to the *NetServer* via a standard Internet Protocol (IP) backhaul interface while end-devices use single-hop wireless communication to one or more gateways. The *NetServer* is responsible for the management of the overall network. For instance, it filters the duplicated packets from different gateways, does a security check, and sends Acknowledgements (ACKs) to the gateways. The nodes in a LoRaWAN network are asynchronous and communicate when they have information to transmit whether event-driven or scheduled. This asynchronous communication is a major driver of the battery lifetime increase. LoRaWan end-devices can serve different applications, each one with its own requirements. In order to optimize a variety of end application profiles, LoRaWAN defines three different device classes (Class A, Class B and Class C). The device classes trade-off network downlink communication latency over the battery lifetime. Besides an improved communication range, the transceivers have unique features derived from the employed modulation schemes. Thus, when creating a network using these transceivers, their capabilities along with the specific network requirements should be taken into account in order to maximize the overall network performance. For instance, in a LoRaWAN network, nodes are not associated with a specific gateway. Instead, data transmitted by a node is typically received by multiple gateways, creating redundancy and the need to filter duplicated packets. Therefore, replacing the LoRaWAN MAC layer while keeping the chirp spread spectrum (CSS) physical layer is an attractive option to develop and evaluate new MAC protocols under this technology.

An example of a replacement for the LoRaWAN MAC layer was proposed by Bor et al. [24]. They designed and implemented LoRaBlink, a MAC protocol to support reliable and energy-efficient multi-hop communication along with low-latency bi-directional communication. The protocol uses time synchronization to define slotted channel access, where the nodes can transmit their data packets. As an important aspect, the authors assume low density, low traffic volume, and a limited number of nodes within the network. Following an evaluation over a network composed of six nodes over an outdoor environment, the authors, by exploring LoRa’s interesting physical capabilities such as its long-range and its non-destructive concurrent transmissions, concluded that LoRa radios (with the physical (PHY) layer) can be used for IoT applications in more general network architectures than LoRaWAN. Table 2 summarizes the main features of the above mentioned LoRa MAC protocols, and correlates them with the multi-gateway MAC LoRa employed in our smart city platform.

### 2.3. DTN Forwarding Strategies

Traditional routing protocols for wired and wireless networks fail to work in challenging environments since they demand a stable end-to-end connection between sources and destinations [25]. Since DTNs suffer from frequent disconnections, in long-duration partitioning with no end-to-end path, routing protocols for this type of networks must adapt themselves to the challenging environment.

Usually, the routing protocols implement a trade-off between controlled replication and network knowledge. A pure replication protocol, e.g. flooding, consumes high resources since the packets are transmitted to all vicinity nodes, hence leading to high network congestion. However, a pure knowledge protocol requires also high resources to process complex routing algorithms and maintain updated routing tables in each node. Thus, it is necessary to find a trade-off between both approaches.

Several studies on DTN routing approaches have been performed over the years. D’souza and Jose [26], Benamar et al. [27], Abdelkader et al. [25], and Sobin et al. [28] presented some of those studies about the overall evolution of the several DTN routing/forwarding strategy approaches. Some forwarding strategies with high relevance to our opportunistic and delay-tolerant network were studied, e.g, Epidemic, the Hybrid of Probability and message Redundancy algorithm, GeoSpray, the Geographical Routing scheme with Angle-Based Decision, Velocity and Direction, and Delegation Query.

The Epidemic [29] protocol is a multi-copy protocol that implements a replication scheme that simply floods the bundle through the network, i.e., it transmits all bundles to all encountered neighbors that have not those bundles already. Thus, it does not require any prior knowledge about the network. In a contact opportunity, the nodes exchange the bundles that they do not have in common. This can be considered the optimal solution in an environment with no memory and bandwidth constraints. Hence, the Epidemic routing protocol minimizes the delivery delay and maximizes the delivery ratio as messages may reach the destination through multiple paths.

The Hybrid of Probability and message Redundancy (HPR) [30] routing algorithm is based on a combination of message delivery probability and message redundancy with the aim of reducing the communication overhead while keeping the high message delivery ratio. This algorithm estimates the delivery probability of the node based on the history of encounter information and contact duration, in order to provide a more precise and reasonable estimation of delivery probability.

GeoSpray [31] is a geographic routing protocol for delay-tolerant networks (DTN) over a vehicular environment. Geographic routing relies mainly on location information and other mobility parameters provided by positioning devices such as global positioning systems (GPS). The GeoSpray routing protocol employs the concept of "spray phase" from the Spray and Wait protocol, where a fixed number of bundle copies are distributed to distinct nodes in the network. However, instead of doing blind replication (as proposed in Spray and Wait), GeoSpray guarantees that bundle copies are spread to the network nodes closer (and/or arrive sooner) to the bundle’s destination. Additionally, the GeoSpray allows each node to forward the bundle copy to another node that can take the data closer to the destination. Therefore, this protocol controls flooding through the settlement of an upper bound on the number of replicas per bundle, while minimizing the transmission overload and resource consumption.

The Geographical Routing scheme with Angle-Based Decision [32] is a geographic awareness routing protocol for message delivery in delay-tolerant networks (DTNs). This scheme can select relay nodes in geographic proximity to perform message delivery towards a destination. It consists of three design functions: (1) inclusion of the advantages of Spray and Wait; (2) consideration of the moving direction, velocity, and angle of each relay node; and (3) the selection of an appropriate relay node which is moving toward the destination. By exploiting geographic locality information, this protocol is able to select an appropriate relay node which is moving towards the destination, and iteratively hands over message copies to relay nodes in a network.

The Velocity and Direction (VeloSent) routing protocol [33] operates in three consecutive phases. First, an analysis of the environment is performed to detect neighboring nodes and their respective contexts. Then, the neighbors that met the destination more recently are considered using the context information about time, location, and velocity of the destination node. Secondly, from this information, an estimation of the new location of the destination node is obtained (a straight line route is considered). In the third phase, the estimated location of the destination is used in order to determine which of the neighboring nodes will most likely meet the destination node. This process is possible by mathematically estimating the timing and the location of a likely encounter between them.

Delegation Query (DelQue) [34] focuses on sources initializing interest-based queries and select relays by considering their capabilities for both query and response. The chosen relays take charge of both querying the relevant interest data and returning it to the demander. DelQue uses geo-community and mobility prediction in its algorithm. The Geo-community concept is used to explore location information from a community, thereby taking advantage of its geolocation. Mobility prediction is used when the source has mobile properties. Spatio-temporal prediction is also used, in order to exploit the information that some nodes obtain at a given location and at a given interval of time.

In order to give a more general view about the trade-offs between the aforementioned routing protocols and our proposed forwarding strategies (Contact-Based Controlled Replication with Neighborhood Classification and Mobility-Based Controlled Replication with Neighborhood Classification), Table 3 summarizes the differences and similarities between them.

## 3. Architecture Overview

**Figure 1:**
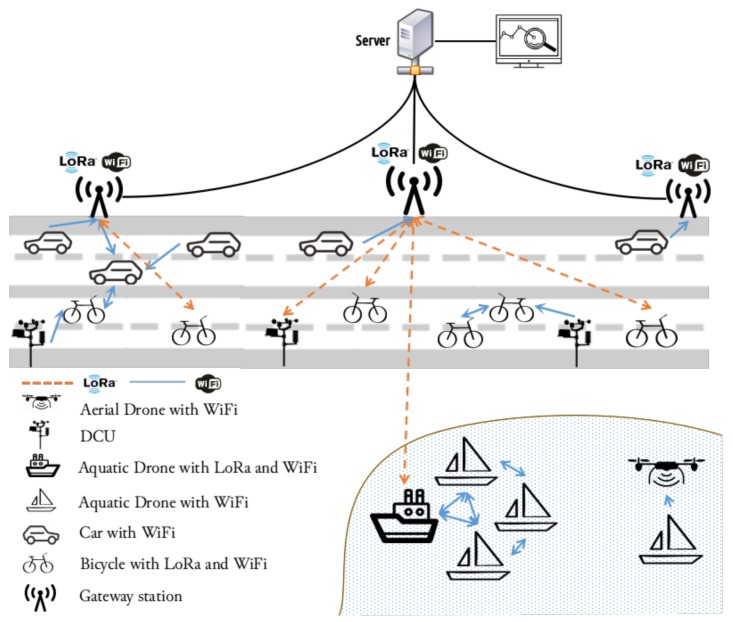
Architecture overview.

Our platform architecture, illustrated in Figure 1, aims to provide a city-wide scenario where heterogeneous elements, such as cars, aerial and aquatic drones, bicycles, or fixed sensors stations, can interact between themselves, either by direct or indirect connections, producing a large, unified and extremely heterogeneous network. With that end, we can identify four different main components that compose our architecture: data-collecting units (DCUs) equipped with monitoring sensors, mobile nodes, gateways, and a server.

In the scope of the IoT paradigm, the communication must allow the seamless integration of any object with the Internet, allowing new forms of interaction between people and devices or directly between devices (machine-to-machine, M2M). In this way, the infrastructure to support the development of an IoT environment must address the following requirements, which are presented in the proposed platform:

- An infrastructure capable of collecting and disseminating a large amount of data through heterogeneous nodes, with the purpose of delivering the information to gateways stations, and therefore, to a database;
- An infrastructure considering multi-technology communication: WiFi for short-range communication, and LoRa, on the other hand, for long-range communications;
- An infrastructure capable of serving as an evaluation platform for a wide range of purposes, going from the different delay-tolerant network (DTN) forwarding decisions to the multi-gateway LoRa MAC protocol.

### 3.1. Network Elements

Each network element (data-collecting units, mobile nodes, or gateways) is based on a central processing unit (Raspberry Pi board) with the hardware specifications described in Table 4.

To achieve the multi-technology communication, allied with the Raspberry Pi embedded WiFi interface, a SX1272 LoRa module manufactured by Libelium is used. In order to establish interaction between the SX1272 LoRa module and the Raspberry Pi, a Multiprotocol Radio Shield (also manufactured by Libelium) must be connected along with the module. This shield will work as a connection bridge between both components. A summary description of the SX1272 LoRa module characteristics is presented in Table 5.

The selected hardware provides a low-cost and easy-to-repair structure.

#### 3.1.1. Data-Collecting Units

Data-collecting units are stations, without wired connectivity to other entities of the network, composed of a set of sensors with the purpose of collecting environmental information. Each DCU is equipped with a large environmental monitoring sensor set that aims to collect relevant information about the environment condition in a dense urban scenario. This sensor set is able to measure the following environmental variables:

Temperature;Luminosity;Wind direction;Wind speed;Carbon dioxide (CO_2_);Sound detection;Humidity;Precipitation;Barometric pressure;MultiGas (CO, CH_4_, NH_3_);UV index.

**Figure 2:**
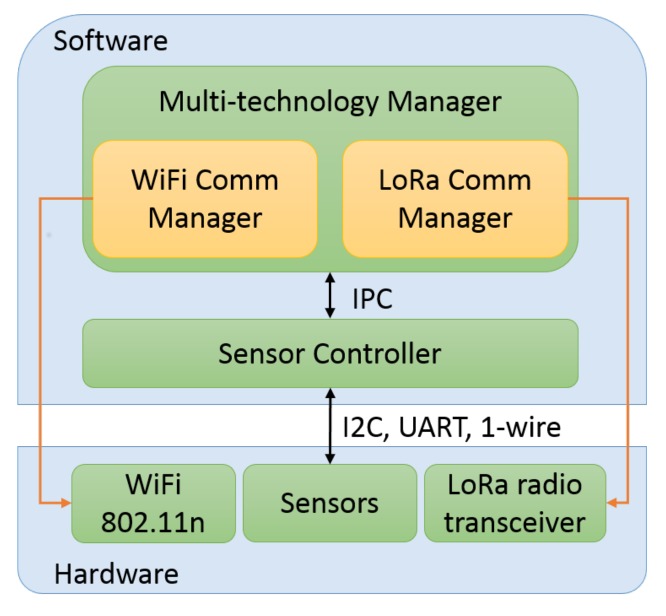
Data-collecting unit (DCU) software architecture. IPC: Inter-Process Communication; UART: Universal Asynchronous Receiver-Transmitter; I2C: Inter-Integrated Circuit.

To sense the city, a DCU must deal with the internal communications of the aforementioned environmental sensors, and gather and store the sensed information while handling the multi-technology capabilities. The DCU software architecture is presented in Figure 2. During its development, both hardware and software were designed with the aim of giving versatility and adaptability to the DCU, so that it can integrate new sensors and functionalities such as the inclusion of new communication technologies.

#### 3.1.2. Mobile Nodes

Mobile nodes are sets of different mobile entities that can comprise bicycles, cars, or drones (aerial or aquatic), increasing the network heterogeneity. Our platform, as already mentioned, focuses on mobile nodes equipped with both WiFi and LoRa communication capabilities. They are not only a fundamental element in the data collection task, since a mobile node interacts with DCUs in order to gather their stored information, but they are also responsible for the data forwarding among mobile elements over an opportunistic delay-tolerant network, where intermediate nodes act as relays in a store, carry, and forward fashion.

**Figure 3:**
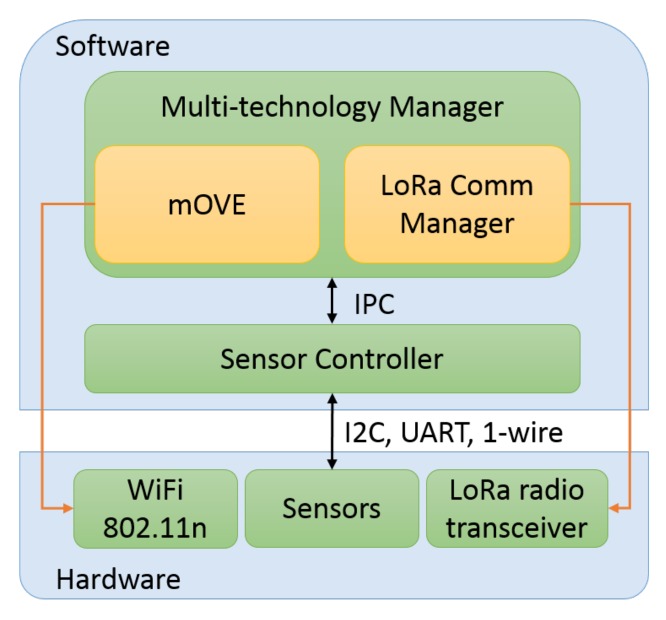
Mobile node software architecture. mOVE: mobile Opportunistic VEhicular.

The multi-technology capabilities allow the mobile nodes to send information to a LoRa gateway, complementing the WiFi opportunistic network with long-range technology, and consequently enlarging the range of applications that the platform can achieve. For instance, applications such as the rescue of DTN expired data packets in order to concede them a new opportunity to be delivered using LoRa communication, or even the periodic dispatch of reports about the mobile node’s geographical location are possibilities enabled by it. Figure 3 shows the mobile node software architecture.

#### 3.1.3. Gateway Stations

Gateway stations are fixed network elements. These nodes are the endpoint of the sensed data packets, meaning that they are the final element of the data gathering plane. A fundamental characteristic of any gateway station is the capability to establish communication to a remote server (the Data Broker), so that it can forward the received information to a database. To achieve this, the gateways have connectivity with a wired network backbone.

**Figure 4:**
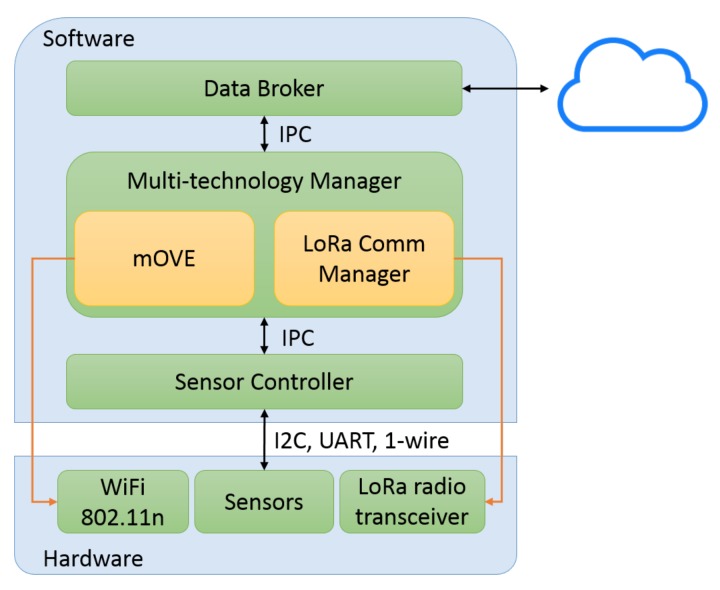
Gateway software architecture.

Due to the multiple technologies, the gateways will act as endpoints for both WiFi and LoRa communications. Regarding the WiFi technology, they are the final element of the implemented opportunistic delay-tolerant network, which means that the intermediate DTN nodes (mobile nodes) relay the information until a gateway is found in its neighborhood. On the other hand, with respect to LoRa technology, gateway stations are the destination for the dispatched information received directly from the DCUs and mobile nodes. Figure 4 shows a gateway station software architecture.

### 3.2. Platform Software Architecture Overview

**Figure 5:**
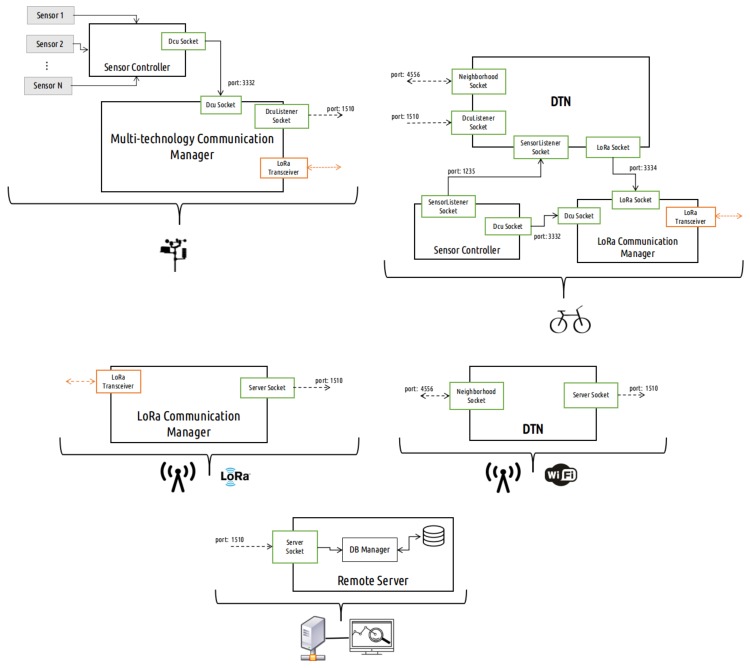
Proposed software architecture overview.

As stated previously, our multi-technology platform addresses different network elements. Figure 5 overviews the platform software architecture of each element, as well as the overall connections established between them in order to achieve our platform requirements. This way, it is possible to better understand the overall complexity between the several network elements and perceive the platform’s intercommunication procedures.

A brief description about the distinct software modules that assemble the platform architecture is now detailed:

**Sensor Controller:** Module responsible for establishing and managing all the direct interactions between the controller board and the element’s inner sensors.
**Multi-technology Communication Manager:** Module responsible for establishing the behavior of each communication interface, WiFi, and LoRa. It has the authority of defining the most suitable technology to deliver its stored data packets (built-in Sensor Controller), according to the information type and the interface availability, until they reach a LoRa gateway or are transfered to a mobile node.
**LoRa Communication Manager:** Module responsible for handling all the behavior regarding the LoRa communication interface. Unlike what happens in a DCU, where we have the Multi-technology Communication Manager, this software module only has to deal with the LoRa technology, since the WiFi is managed under the DTN operating processes.
**DTN:** Module responsible for implementing the disrupted and delay-tolerant network architecture.

## 4. Multi-Technology Communication

Multi-technology exploitation has the purpose of providing a more resourceful and flexible architecture, since it allows for covering some technology inconsistencies with another form of communication technology. LoRa and WiFi were the chosen technologies to be employed over the proposed scenario, mainly because they present distinct capabilities in terms of connectivity range and bit rate, among others. Thus, besides the implementation of a LoRa network, it was decided to configure the platform with an additional urban sensor network based on a DTN. The DTN will be supported by the WiFi technology, namely IEEE 802.11b/g/n.

**Figure 6:**
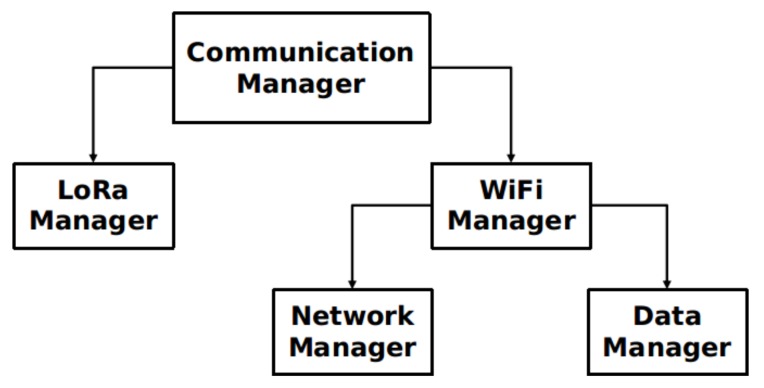
Multi-technology communication overview.

With the multi-technology communication, an agent had to be designed in order to manage the behavior of the communications. The definition of when, how, and which technology should dispatch the data are some of the tasks under its responsibility. In order to achieve such goal, several sub-modules were implemented, namely the WiFi Manager and LoRa Manager. Figure 6 gives an overview of our multi-technology communication manager organization by giving the hierarchical design of the several implemented sub-modules.

***WiFi Manager*:** This sub-module is responsible for managing the WiFi interface and how its connection should be handled. It performs an active WiFi scan, in order to discover any mobile node (DTN node) in its vicinity.
   **Network Manager:** This sub-component searches and identifies mobile nodes within a DCU’s vicinity.
   **Data Manager:** This sub-component manages how the waiting data is handled when the DCU has an available destination to forward the data.
***LoRa Manager*:** This sub-module is responsible to handle the LoRa communication interface.

### 4.1. Medium Access Communication Protocol for LoRa Radio Technology

In radio communications, a robust MAC protocol has to exist to coordinate the medium access in an effective way, especially in the case of multiple gateways, which is the case of this work. Thus, our platform employs the multi-gateway LoRa MAC protocol developed in [36], which follows an approach based on carrier sense multiple access with collision avoidance (CSMA/CA) with Request To Send (RTS)/Clear To Send (CTS) message exchange to control the medium access of the devices. Moreover, influences from protocols such as the multiple access with collision avoidance (MACA), multiple access with collision avoidance for wireless (MACAW), and the IEEE 802.11 MAC layer can be found.

### 4.2. DTN Forwarding Strategies

Developed by the Network Architectures and Protocols (NAP) research group (https://www.it.pt/Groups/Index/62), mOVE [37] is a DTN-based architecture supported by the conventional WiFi technology IEEE 802.11a/b/g. Each node receives information from other DTN nodes, stores it (on a persistent storage device), and forwards it according to the neighborhood availability, exploiting a vehicle-to-vehicle (V2V) and a vehicle-to-infrastructure (V2I) multi-hop based communication, leveraging upon the V2X concept. mOVE is implemented in C/C++ programming language and it is designed to be highly modular and extensible. It can be used to develop a large set of applications which rely on delay tolerant communication using vehicles (or other mobile elements) as carriers of information.

The versatility achieved through the proposed architecture grants the possibility to perform a miscellaneous set of tests and evaluations. One of which is the performance evaluation of DTN forwarding schemes. In order to evaluate proposed forwarding strategies, some "classic" DTN decision schemes were implemented, more specifically, the *Epidemic* strategy, which is a flooding-based protocol that aims at the maximization of the delivery ratio and the minimization of the end-to-end delay without considering the limitations of network resources. As a result, several constraints, such as the nodes’ buffer size limitation, restricts the practical performance of this protocol.

With the aim to increase the delivery ratio while minimizing the network resources consumption, two forwarding/decision strategies were proposed and evaluated over our platform:

- Contact-Based Controlled Replication with Neighborhood Classification;
- Mobility-Based Controlled Replication with Neighborhood Classification.

Due to the characteristics of the proposed platform, such as the high mobility pattern that an opportunistic network presents over a smart city scenario, a high number of contacts between neighbors is expected. Thus, the proposed Contact-based Controlled Replication with Neighborhood Classification strategy estimates a node delivery probability by relating the number of past contacts and the time elapsed since the last encounter with a gateway, instead of using the contact duration and encounter frequency as proposed by the Hybrid of Probability and message Redundancy strategy [30]. On the other hand, different approaches take advantage of the mobility parameters of each neighbor in order to evaluate which one offers a better probability of reaching the destination first, e.g., the GeoSpray [31] and Geo-Routing with Angle-Based Decision [32] strategies. However, unlike these strategies, the proposed Mobility-Based Controlled Replication with Neighborhood Classification estimates a node delivery probability based on the mean velocity and the angle between the forwarder and the receptor node without having the knowledge about the destination location.

It is of high importance to understand which neighbors should receive the data packets, i.e., a mobile node should be able to evaluate its vicinity nodes to find the best neighboring node for data forwarding. For this, each node collects personal information and exchanges it with its neighbors through Neighbor Announcement packets. As stated previously, two different vicinity classification approaches were developed, resulting in two forwarding approaches. It follows a brief explanation about each decision strategy, Contacts-Based and Mobility-Based, respectively:

**Contacts-Based Neighborhood Classification:** This evaluation process classifies a mobile node according to the following information: (1) the number of contacts that occur over a predefined period of time, and (2) the last recorded *timestamp* in which a node had contacted a gateway station. With this information a classification table is computed as follows
  (1) $$Rank=W_{Last}\cdot P_{lastRSU}+W_{C}\cdot P_{Contact},$$
  where $W_{Last}$ represents the weight given to $P_{lastRSU}$, and $W_{C}$ represents the weight given to $P_{Contact}$, which are 1/2 unless otherwise specified. $P_{lastRSU}$ represents the probability of reestablishing contact with a road side unit (RSU), which is given by
  (2) $$P_{lastRSU}=1-\frac{time_{actual}-time_{lastRSU}}{time_{actual}},$$
  and $P_{Contact}$ relates to the mobility of the node and the number of contacts with RSUs or other mobile nodes in a previous time window, and is given by
  (3) $$P_{Contact}\left(N_{Contact}\right)=\left\{\begin{array}{cc} 0, & N_{Contact}=0\\ \frac{N_{Contact}}{\tau_{Contact}}, & 0\leq N_{Contact}\leq \tau_{Contact}\\ 1, & \tau_{Contact}<N_{Contact} \end{array}\right.$$
  where $\tau_{Contact}$ defines an acceptable number of contacts per time window that a mobile node should contact to be considered a *good* neighbor.
**Mobility-Based Neighborhood Classification:** This evaluation process classifies a mobile node according to the following information: (1) the node mean velocity, and (2) the node heading angle. With this information a classification table is computed as follows:
  (4) $$Rank=W_{Head}\cdot P_{Heading}+W_{Vel}\cdot P_{MeanVel},$$
  where $W_{Head}$ represents the weight given to $P_{Heading}$, and $W_{Vel}$ represents the weight given to $P_{MeanVel}$. $P_{Heading}$ relates to the difference on the heading angle between the node and its neighbors which is given by
  (5) $$P_{Heading}=\frac{|heading_{node1}-heading_{node2}|}{180},$$
  and $P_{MeanVel}$ relates to the mobility of the neighboring nodes, and is given by
  (6) $$P_{MeanVel}\left(meanVel\right)=\left\{\begin{array}{cc} 0, & meanVel=0\\ \frac{meanVel}{\tau_{Vel}}, & 0\leq meanVel\leq \tau_{Vel}\\ 1, & \tau_{Vel}<meanVel \end{array}\right.$$
  where $\tau_{Vel}$ defines an acceptable value of mean velocity that a mobile node should perform to be considered a *good* neighbor. The usage of the heading angle is employed with the aim to identify nodes traveling in opposite directions.

Both forwarding strategies employ two network control mechanisms: loop avoidance and the congestion minimization. Scenarios where a data packet is continually routed through the same nodes are undesirable, mainly because a significant amount of the network resources are consumed while performing redundant actions. To solve this issue, the proposed forwarding scheme resorts to the data packet tracking information, i.e., to the list of previous hops (previous nodes). Limiting the number of hops that a packet can travel grants the possibility of controlling the impact of having the list of traveled hops information within the packet header, allowing the application of the loop avoidance mechanism without compromising the network scalability.

High congestion has a direct impact on the network performance and on the data dissemination process. In order to prevent and overcome this problem, a congestion minimization technique is employed. As previously described, the information on the packet’s number of hops gives feedback about the data packet depth within the network. Therefore, an estimation of the overall packet distribution in the network can be made using the number of hops information. When the number of hops is high, it means that the packet is stored in several nodes, which means that the packet is spread over the network. On the other hand, when the number of hops is low, it means that the packet reaches only a few number of nodes and does not have a significant distribution over the network. Therefore, the proposed technique enables the sending decision on the packets with a minor presence over the network. Algorithm 1 summarizes the forwarding procedure.

**Algorithm 1** Forwarding algorithm  1: **procedure**
Forwarding Decision(decision
logic)  2:     **while** Packets *p* in storage **do**  3:         p←peak packet  4:         **if** neighbors neigh available **then**▹ with Loop Avoidance  5:            $p_{sending}\leftarrow$ ProbabilityFuntion($p_{hops}$)  6:            **if**
$p_{sending}>rand\left(\right)$
**then**▹ with Congestion Minimization  7:                neigh← getBestNeighbor(ownRank)▹ with Neighborhood Classification  8:                **if**
neigh≠NULL
**then**  9:                    send packet *p* to neighbor neigh10:         **wait**
cycle
delay

## 5. Results

The developed smart city platform framework envisions a citywide deployment over the city of Aveiro. Making use of the developed network elements and the multi-technology employment, it leverages the multi-gateway MAC in the LoRa protocol in order to ensure reliable communications within this technology, and the WiFi opportunistic and delay-tolerant network to increase the heterogeneity of the platform.

Versatility and adaptability denote core characteristics of the described platform. Its implementation endows it with a modular design capable of integrating new features, e.g., new communication technologies, additional sensory equipment, new network functionalities, and new mobile entities, conferring a dynamic nature to it. Therefore, heterogeneity is a key aspect of the platform, since it is able to cope with distinct network elements and contrasting features, for instance the distinct speeds of mobile nodes. Such properties allow the deployment of a large variety of smart city applications. Being a smart city platform, scalability was taken into consideration during its design and implementation. A real use case scenario was used to evaluate our smart city multi-wireless technology with an opportunistic communications platform.

### 5.1. Use Case Description

Ria de Aveiro (the Aveiro lagoon) is an ex-libris of this city. With a large population of inhabitants in the watershed area, it has a considerable regional and national economic importance, namely through the activities related to: port facilities, industries, aquaculture, salt-production, fishing, tourism, recreational activities, and agriculture. Regarding tourism, the moliceiro tours that go through the lagoon channels within the city heart are appealing touristic activities for those who travel to Aveiro.

**Figure 7:**
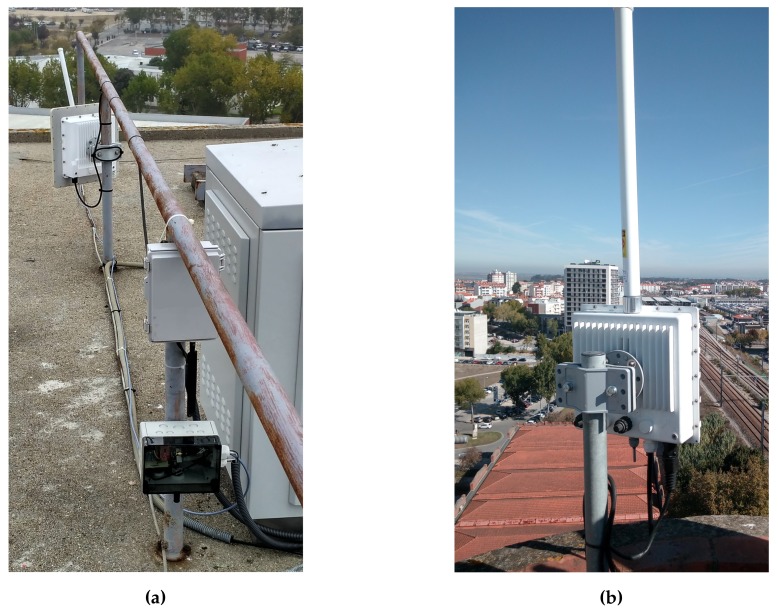
LoRa gateway deployment (**a**) LoRa gateway deployed in the University of Aveiro; (**b**) LoRa gateway deployed in Aveiro’s Cultural and Congress Center.

As the starting point of our platform deployment over the city of Aveiro, two fixed LoRa gateways were strategically placed over two separated locations with a high view over a broad city area, with the objective of achieving good city coverage through this communication technology. Figure 7 shows this deployment.

**Figure 8:**
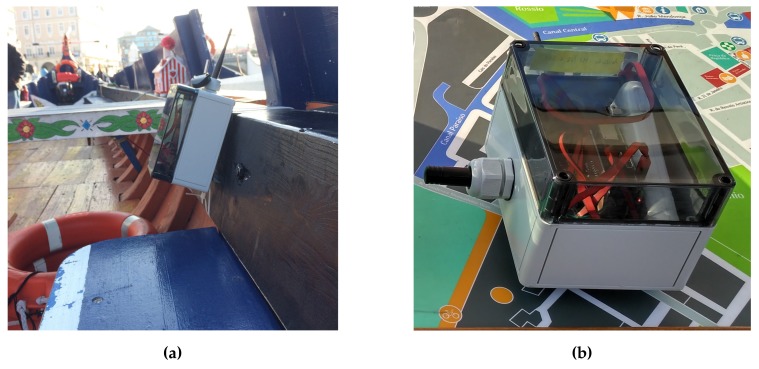
DCU deployment (as mobile nodes) (**a**) A mobile DCU deployed in a moliceiro; (**b**) Closer look of a mobile DCU.

**Figure 9:**
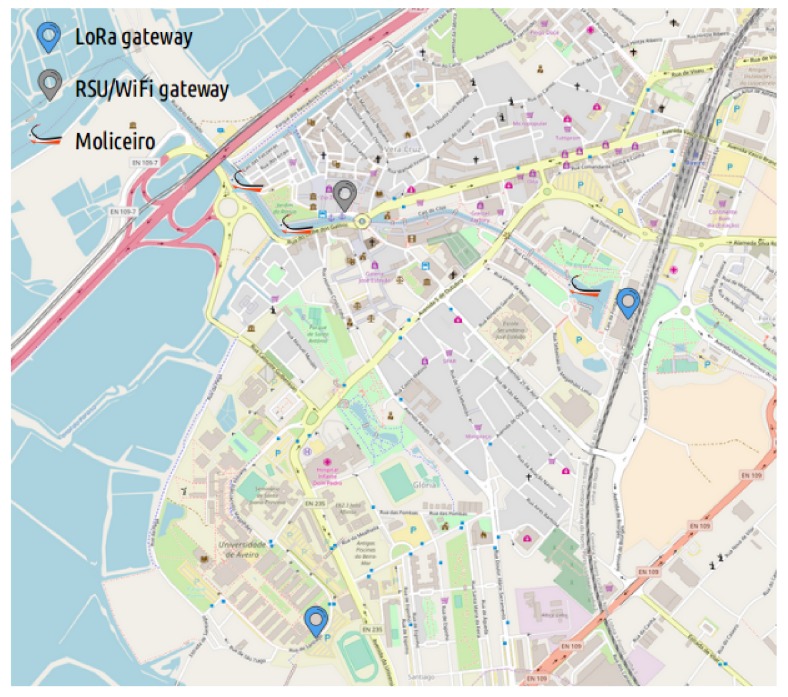
Use case scenario topology.

With the LoRa communication assured by the gateways already installed, we took advantage of the developed DCUs and the mobile nodes software architecture to endow three moliceiros with sensory data acquisition and multi-technology communication capabilities, as shown in Figure 8. The collected data holds information about the node’s geographical location and the sensory measurements from its own sensors. In order to further evaluate our multi-technology platform, we installed one RSU/WiFi gateway in a dock where some moliceiros wait for the arrival of tourists to start their cruises. The use case’s overall deployment and network topology are depicted in Figure 9.

The use case evaluation comprehends three consecutive days of February 2018. For each day, a distinct forwarding strategy over the delay-tolerant network that our platform considers was evaluated, namely Epidemic, Contacts-Based Controlled Replication with Neighborhood Classification, and Mobility-Based Controlled Replication with Neighborhood Classification. It is important to keep in mind that the routes and the number of trips made by each moliceiro are not guaranteed to be the same over the different evaluation days, as these factors depend on the availability of each moliceiro’s captain and the number of tourists.

We present an evaluation scenario to test our multi-technology opportunistic smart city platform using a real deployment and a real application over moliceiros cruising the city of Aveiro. Some considerations and general characteristics regarding the experiments are as follows:

- The network is composed of three moliceiros, one WiFi gateway, and two LoRa gateways.
- The positions of the gateways are fixed as shown in Figure 9.
- The LoRa communication interface is used to transmit information about the node’s geographical location only. The packet’s transmission rate follows the imposed regulatory duty-cycle constraints (1% duty-cycle, i.e., 36 seconds per hour).
- Each moliceiro generates data from the measurements of its own sensors with an acquisition period of six seconds;
- The WiFi communication interface is managed by the already described opportunistic delay-tolerant network (mOVE). The mOVE storage will be populated with the sensed data packets.
- The evaluation lasted three consecutive days, in which Epidemic, Contacts-Based Controlled Replication with Neighborhood Classification, and Mobility-Based Controlled Replication with Neighborhood Classification were evaluated, respectively.
- Each strategy was evaluated during a day of experiment with total duration of seven hours (10 a.m. to 5 p.m.).

### 5.2. Network Topology Evaluation Per Day

**Figure 10:**
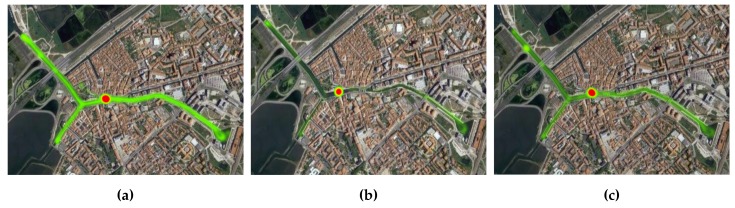
Moliceiros flow over day 1—Epidemic strategy evaluation (**a**) Moliceiro-101 flow; (**b**) Moliceiro-102 flow; (**c**) Moliceiro-103 flow.

**Figure 11:**
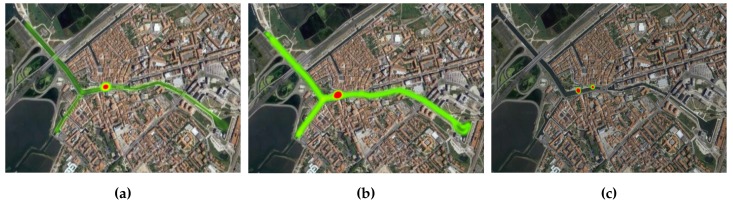
Moliceiro flow over day 2—Contacts-based Controlled Replication with Neighborhood Classification strategy evaluation (**a**) Moliceiro-101 flow; (**b**) Moliceiro-102 flow; (**c**) *Moliceiro*-103 flow.

**Figure 12:**
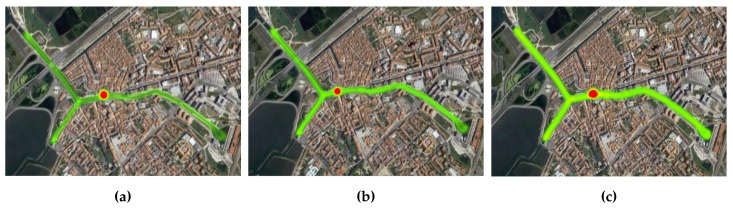
Moliceiros flow over day 3—Mobility-Based Controlled Replication with Neighborhood Classification strategy evaluation (**a**) Moliceiro-101 flow; (**b**) Moliceiro-102 flow; (**c**) Moliceiro-103 flow.

Having already established the overall scenario topology, this subsection presents an evaluation of the mobility pattern that each moliceiro presented over the several days of experimentation. This evaluation will allow us to observe the contrasts and dissimilarities between the mobility flows of each moliceiro within the same evaluation day and between different evaluation days. Figure 10, Figure 11, and Figure 12 present these results.

This analysis grants an important characterization of each mobile node mobility pattern. With this, conclusions about the number of tourists, the number of trips made by each moliceiro, and the geographical areas populated by the moliceiros, among others, can be drawn. Consequently, important characteristics regarding the overall network behavior during the evaluations can also be made:

- The red spot over the different heat maps represents the stopping points/docks where the moliceiro stops before the next trip.
- Considering the first day, node-101 presents a better activity pattern. This is also the case for node-102 during the second day and finally for the node-103 during the last evaluation day.
- During the second day of evaluations, node-103 did not travel;
- The third day of evaluation presented with better overall activity with respect to all moliceiros, particularly node-103.

### 5.3. Network Analysis

The following subsections aim to characterize and portray the performance of our multi-technology opportunistic platform through: (1) the performance analysis of the implemented multi-gateway LoRa MAC protocol; and (2) the behavioral examination of the opportunistic and delay-tolerant network (mOVE) considering the previously proposed forwarding strategies.

**Figure 13:**
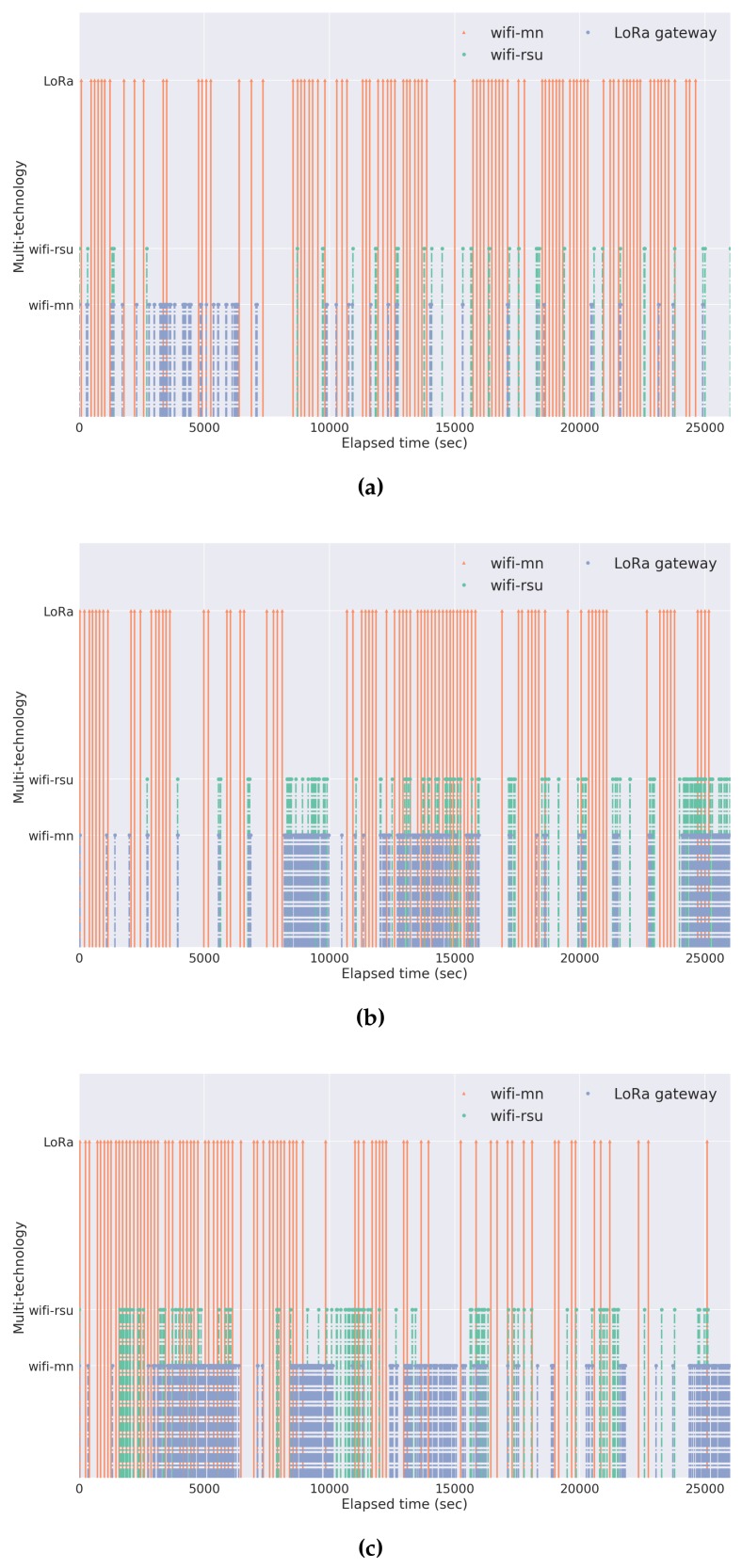
Multi-technology map over day 2 (**a**) Moliceiro-101; (**b**) Moliceiro-102; (**c**) Moliceiro-103.

Keeping in mind the dissimilarities of the network nodes’ mobility patterns between the evaluations and to better understand the network behavior, namely its multi-technology capabilities, a multi-technology map from one of the evaluation days was arbitrarily chosen. Figure 13 presents the multi-technology map from the second day of experiments. This map represents:

- The temporal instant in which each node achieved a successful transmission of a data packet through LoRa technology; and
- The temporal instant in which each node entered in the WiFi neighborhood of other network nodes (moliceiros or RSU).

This multi-technology evaluation allows us to observe the overall performance of each node’s connectivity. For the LoRa technology, the noticeable lack of periodicity in sending the node’s geographical information to the gateway is due to the lack of connectivity between the network nodes and the LoRa gateways. An improvement in order to handle the connectivity issues would be the change of the LoRa communication mode employed in this evaluation. This mode can be changed to a different one with a lower transmission rate in favor of higher achievable ranges. Also, it is important to notice that these evaluations were performed over a dense urban center, which is more susceptible to the loss of a communication link when compared to rural fields. On the other hand, regarding the WiFi communications, the interactions between the several network nodes are noticeable. These interactions can be shorter, in the case of two moliceiros crossing paths, or longer, if both nodes are stopped within the range of each other.

The co-existence of simultaneous LoRa and WiFi communications results from the fact that our multi-technology platform allows for the sending of differentiated data from each technology interface, increasing the platform’s connectivity. Since we are taking advantage of two communication technologies with different radio frequency physical layers (different operating frequencies), it is ensured that there is no radio interference between them.

#### 5.3.1. LoRa MAC evaluation

To evaluate the performance and behavior of the implemented LoRa MAC protocol, the obtained results for each day of experiments are presented. They express, respectively, an analysis on: the data packet delivery ratio, the average number of attempts per channel access, and the backoff time used per node.

**Figure 14:**
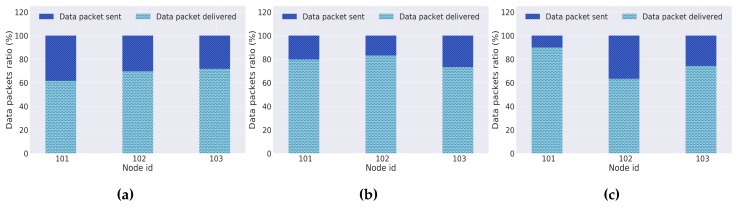
LoRa’s data packet delivery (**a**) Day 1; (**b**) Day 2; (**c**) Day 3.

The results for the overall data packets delivery ratio through LoRa technology for each day of experiments are shown in Figure 14: they represent the percentage of data packets that each network node delivered successfully to the gateway after having acquired the medium access. As previously stated, the geographical location where the nodes spend most of its time, the docks, have a noticeable lack of LoRa connectivity to the gateways. A proposal to solve this obstacle has already been presented. Besides, during a moliceiro cruise, various obstacles (such as bridges) can impose the lack of communication or an unexpected loss of packets through this technology. Such conditions led to low delivery rates by the LoRa technology (between 60% and 85%). However, due to our platform’s multi-technology capabilities, the information that was not delivered by LoRa will be recovered and forwarded through the opportunistic and delay-tolerant network.

**Figure 15:**
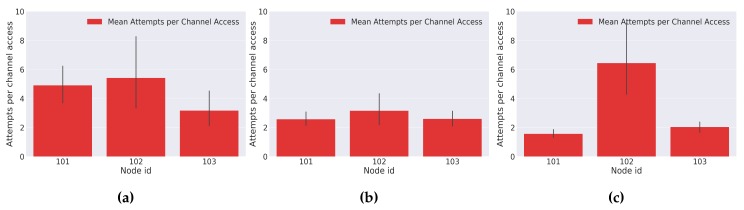
LoRa’s mean attempts per channel access (**a**) Day 1; (**b**) Day 2; (**c**) Day 3.

The results for the overall number of attempts to gain the channel access are presented in Figure 15. They represent the mean amount of attempts made by each node until it was granted with the medium access. A further analysis of the results shows that in general, nodes presenting higher number of attempts per channel access are also the ones with poorer data packet delivery ratios. Therefore, it is concluded that these nodes suffered a lack of a stable communication link between them and a LoRa gateway. Once more, we would like to emphasize that the high mean number of channel accesses attempts presented by the network nodes can be solved and reduced by the introduction of a novel LoRa gateway with a geographical location closer to the city area where the evaluations were performed, in order to guarantee better communication links between the moliceiros and the LoRa gateways.

**Figure 16:**
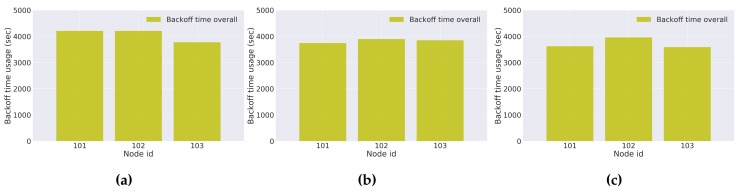
Overall backoff usage (**a**) Day 1; (**b**) Day 2; (**c**) Day 3.

The results for the overall backoff time used by each node are presented in Figure 16. They represent the backoff time that each network node had to experience during the experimental period. The high backoff time used by each node reflects the large experimental period per day (seven hours), as well as the number of channel accesses and data packet delivering failures. Therefore, scenarios capable of providing better communication links between the network nodes and the LoRa gateways would increase the data packet delivery rate and decrease the mean number of channel access attempts, resulting in a significant decrease in the backoff time used by each node.

#### 5.3.2. Mobile Opportunistic Vehicular Evaluation

**Figure 17:**
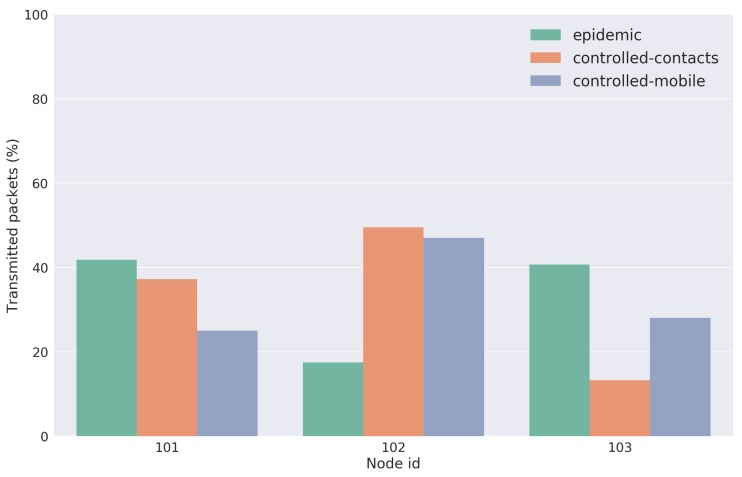
Results of overall transmitted data packets to other mobile nodes.

For the evaluation of the opportunistic delay-tolerant network (mOVE architecture), the obtained results for each evaluated strategy are presented. They show an analysis of the overall transmitted data packets to other mobile nodes; the delivery ratio; the cumulative end-to-end (E2E) delivery delay; and the total network overhead. The results for the overall transmitted data packets within the opportunistic network for each evaluated forwarding strategy are presented in Figure 17. They represent the percentage of data packets that each network node transmitted to others.

Through the analysis of the results, we can conclude that mobile nodes with fewer transmitted packets to other mobile nodes are the ones that presented a poorer mobility pattern, i.e., the ones that performed no or fewer trips. A smaller number of trips implies less frequent contacts with the other network nodes, leading to smaller numbers of transmitted packets. This fact was observed in node-102, for the Epidemic strategy (day 1), in node-103 for the Contacts-Based Controlled Replication with Neighborhood Classification (day 2), and finally in node-101 for the Mobility-Based Controlled Replication with Neighborhood Classification strategy (day 3). Such premises can be confirmed by observing the flow of each network node in Figure 10, Figure 11, and Figure 12.

**Figure 18:**
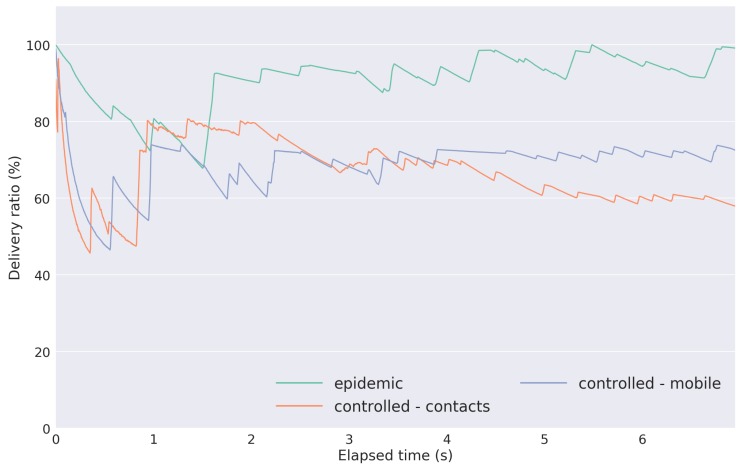
Delivery ratio results.

The results with respect to the delivery ratio for each evaluated strategy are shown in Figure 18. They represent the ratio between the delivered data packets to a gateway and the overall data packets within the network.

As expected, the Epidemic has the highest delivery ratio. Since the Controlled Replication with Neighborhood Classification strategies carry out a trade-off between the amount of network replication and network congestion, a lower delivery ratio allied with a lower network overhead was expected as compared to the Epidemic method. Both strategies (contacts-based and mobility-based) presented similar behaviors (with a final delivery ratio difference of approximately 15%). Part of that difference is due to the distinct mobility patterns that each node presented between the two evaluation days.

**Figure 19:**
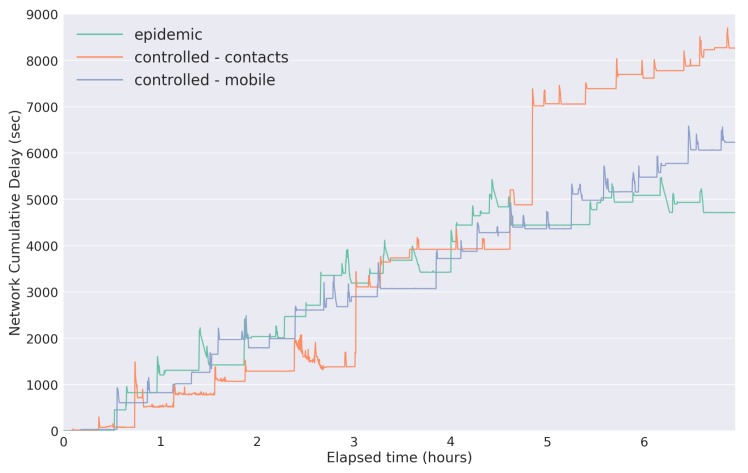
Cumulative network E2E delay results.

The results with respect to the cumulative end-to-end delivery delay for each evaluated strategy are shown in Figure 19. They represent the amount of time that a data packet takes from its generation until it has reached a gateway.

As expected, the strategies with lower delivery ratios are the ones with higher delivery delay. The overall high delivery delay presented by the network is due to the fact that the presented results consider seven hours of evaluation, and there is a short period of data acquisition, specifically one packet every six seconds. The short sensing period was selected to allow us to analyze the behavior and performance of the network with considerable amounts of information flowing.

**Figure 20:**
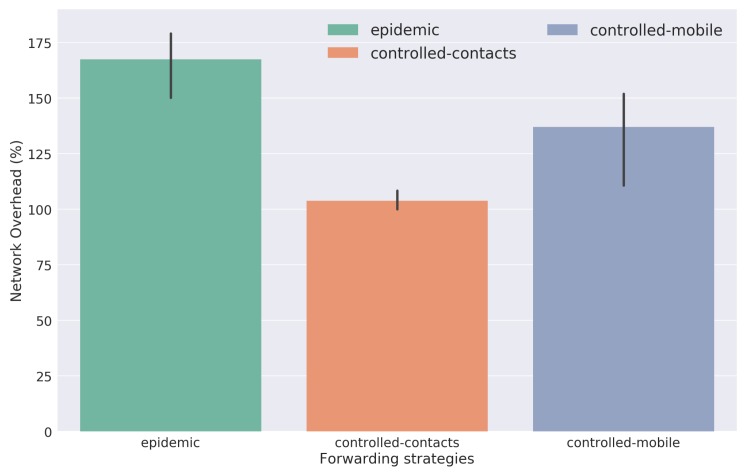
Network overhead results.

The results with respect to the network overhead for each evaluated strategy are presented in Figure 20. They show the number of redundant data packets that each strategy introduces in the network. As expected, the Epidemic strategy introduces the highest network overhead due to its blind replication process without any limitations. Regarding both Controlled Replication with Neighborhood Classification strategies, it is observed that the contacts-based method has a lower network overhead. This was expected since this strategy presented a poorer network node mobility pattern, leading to a less data packet replication and consequently, a lower network overhead.

## 6. Conclusions

Our aim was to develop, implement, and evaluate a multi-technology opportunistic platform for environmental data gathering over smart cities. To that end, this work provided the design and the development of each component constituting the platform: the several network elements, the implemented LoRa MAC protocol, and the proposition of two urban-centric forwarding strategies for an opportunistic and delay-tolerant network.

For the platform’s evaluation we resorted to a real application scenario over the city of Aveiro. Considering moliceiros as the mobile entities of our multi-technology opportunistic platform, we were able to evaluate our platform’s multi-technology capabilities over three consecutive days, with an acquisition of gathered information for a total period of more than 20 h. Several important elements were considered during this evaluation: the multi-technology concurrency (between LoRa and WiFi); the performance and behavior of the medium access control protocol for LoRa; and the performance and behavior of the opportunistic and delay-tolerant network considering our proposed forwarding strategies.

The obtained results showed that, regarding the LoRa technology, we had some losses of packets through this communication interface. However, those losses were not due to poor performance of our LoRa MAC protocol, but to the existence of geographical areas that belong to the moliceiro’s routes where there is a lack of connectivity to any of the LoRa gateways that we had already installed in the city. On the other hand, regarding the WiFi and the opportunistic and delay-tolerant network, we evaluated our two proposed forwarding strategies: Contacts-Based Controlled Replication with Neighborhood Classification and Mobility-Based Controlled Replication with Neighborhood Classification. These strategies’ evaluations showed that the introduced mechanisms, loop avoidance and congestion minimization, were capable of reducing the network overhead (and thus, the network resources consumption) through the prevention of packet loops and through more selective replication based on the past history of the packet traveled hops, respectively. Also, the neighborhood classification mechanisms allowed the replication of data packets only for the most qualified neighbors. With a more selective choice of the next hop, lower network congestion is achieved without compromising the delivery ratio.

Therefore, we were able to implement and assess our smart city platform over a real application scenario. As future steps we aim to further assess our platform with different real application scenarios, over larger evaluation periods and considering a more dense and populated network.

## Figures and Tables

**Table 1:** An overview of IoT platforms for smart cities.

IoT Platforms	Multi-Technology	Long Range	Short Range	Multi-hop	Pros	Cons
[11]	*	*	*	*	Highly-populated network. Multiple communication technologies.	Lacks a low-cost and long-range network. Lacks an opportunistic network.
[14]	*	*	*	*	Multiple communication technologies.	Lacks an opportunistic network.
[16]	*	*	*	*	Multi-hop capabilities with buses s data forwarders.	Lacks low-cost and long-range network.
[17]	-	*	-	-	Extensive LoRa experimental network setup.	Lacks multi-technology, multi-hop and opportunistic communication capabilities.
[18]	-	*	-	-	Mobile sensing (through trams).	Lacks multi-technology, multi-hop and opportunistic communication capabilities. Lacks a low-cost and long-range network.
[19,20]	-	*	-	-	Energy-efficient management.	Lacks multi-technology, multi-hop and opportunistic communication capabilities.
Our proposal	*	*	*	*	Multiple communication technologies. Mobile sensing. Opportunistic and delay-tolerant communications, providing a multi-hop ata forwarding approach.	Still a small-sized network, currently under expansion.

**Table 2:** LoRa medium access communication (MAC) protocol overview. RTS: Request To Send; CTS: Clear To Send; CCTS: Control CTS.

MAC Protocol	Single-Gateway vs. Multi-Gateway	Single-Channel vs. Multi-Channel	Media Access	Network Type	Communication Flow
LoRaWAN	single	multi	Aloha + LBT	single-hop	bi-directional
LoRaBlink	single	single	Slotted Aloha	multi-hop	bi-directional
Multi-gateway LoRa	multi	single	RTS/CTS/CCTS	single-hop	bi-directional

**Table 3:** Forwarding strategies overview.

Algorithms	Type	Single/ Multiple Copy	Replication Rate	Information Needed	Objectives/Comments
Direct Contact	Direct	S	None	N/A	Source moves and delivers the bundle directly
Epidemic	Flooding	M	Very High	N/A	Rapid propagation of data
HPR	Probabilistic	M	N/A	N/A	Messages sent to increasing delivery probability nodes using mecanisms that reduce network overhead
GeoSpray	Geo	S/M	Medium	Navigation	Does not tackle mobile destination
VeloSent	Movement Prediction	M	N/A	Location and velocity associated with time	Tries to predict destination movement, routing accordingly
DelQue	Social	M	Medium	Spatio-temporal mobility	Receiver-driven approach
Controlled Replication - Node contacts	Opportunistic	M	Low	Number of contacts, Timestamps of last connection with the gateway	Uses neighborhood rank classification based on node contact features in a recent time window
Controlled Replication - Mobile	Opportunistic	M	Medium	Mean velocity, heading angle	Uses neighborhood rank classification based on movement properties of nodes

**Table 4:** Raspberry Pi 3 Model B specifications.

**Processor**	1.2 GHz 64-bit quad-core ARMv8 CPU
**Memory RAM**	1 GB
**WiFi Networking**	2.4 GHz 802.11n Wireless LAN
**Operating System**	64-bit Raspbian GNU

**Table 5:** LoRa technology hardware description [35].

**Module**	SX1272
**Dual Frequency Band**	863–870 MHz (Europe); 902-928 MHz (US)
**Transmission Power**	25 mW
**Sensitivity**	–134 dBm
**Channels**	8 (868 MHz); 13 (900 MHz)
**Range**	Line-of-Sight = 21 km; Non-Line-of-Sight = +2 km
